# A type A aortic dissection missed by non-cardiac gated contrast-enhanced computed tomography due to an aortic root dissection flap masquerading as an aortic valve apparatus: a case report

**DOI:** 10.1186/1752-1947-7-285

**Published:** 2013-12-30

**Authors:** Karan Nagra, Richard Coulden, Michael Sean McMurtry

**Affiliations:** 1 1-002 Katz Group Centre for Pharmacy and Health Research, University of Alberta, 8440 112th Street, Edmonton, AB T6G 2E1, Canada; 2 Department of Radiology and Diagnostic Imaging, Faculty of Medicine and Dentistry, University of Alberta, 2C2 WMHSC 8440 112th Street, Edmonton, AB T6G 2B7, Canada; 3 Department of Medicine, Faculty of Medicine and Dentistry, University of Alberta, 2C2 WMHSC, 8440 112th Street, Edmonton, AB T6G 2B7, Canada

**Keywords:** Aortic dissection, CT angiography, EKG gating, Transesophageal echocardiography

## Abstract

**Introduction:**

Though computed tomographic angiography has very high sensitivity and specificity to diagnose acute aortic dissection, false-negative studies can occur and secondary tests may be required to make the diagnosis.

**Case presentation:**

We report the case of a 57-year-old Caucasian man with a typical presentation for acute type A aortic dissection in whom the initial non-cardiac gated computed tomographic angiogram was negative, leading to a delay in surgical management. Transesophageal echocardiography and *post hoc* 3D reconstruction of the original computed tomographic scan revealed a dissection flap confined to the aortic root, immediately superior to the sinuses of Valsalva and masquerading as part of the aortic valve apparatus.

**Conclusion:**

This case demonstrates that false-negative computed tomographic angiograms taken to rule out type A aortic dissection can occur and that secondary imaging tests, such as echocardiography, should be performed in cases in which the pre-test probability of aortic dissection is high. Cardiac gating of computed tomographic angiograms to exclude aortic dissection may enhance diagnostic accuracy.

## Introduction

Acute type A aortic dissection is a life-threatening cardiovascular disorder in which early diagnosis is key to definitive surgical management and patient survival. Though computed tomographic (CT) angiography has very high sensitivity and specificity to aid in diagnosing acute aortic dissection, false-negatives can occur. In this report, we describe the case of a patient with a type A aortic dissection confined to the aortic root that masqueraded as aortic valve apparatus on initial gated non-cardiac CT angiography, but was correctly identified by transesophageal echocardiography.

## Case presentation

A 57-year-old Caucasian man with a smoking habit who had a history of hypertension and dyslipidemia as well as a family history of thoracic aortic dissection presented to a community hospital complaining of sudden onset of chest discomfort and dyspnea. He was diaphoretic, with blood pressure of 114/60mmHg and no pulse deficit, heart rate of 114 beats per minute and respiratory rate of 20 breaths per minute. In addition, he required oxygen by nasal cannula to maintain oxygen saturation above 90%. He had a large-volume central pulse, no extra heart sounds and soft systolic and early diastolic murmurs over the aortic valve. His jugular venous pressure was 4cm above the sternal angle, and he had crackles at both lung bases. There were no features suggestive of Marfan syndrome or connective tissue diseases. The initial emergency work-up included an electrocardiogram (EKG), which showed left ventricular hypertrophy with repolarization abnormalities; a chest X-ray with increased interstitial markings suggesting acute pulmonary edema; a troponin I level of 0.41μg/L; and a brain natriuretic peptide level of 487ng/L.

**Figure 1:**
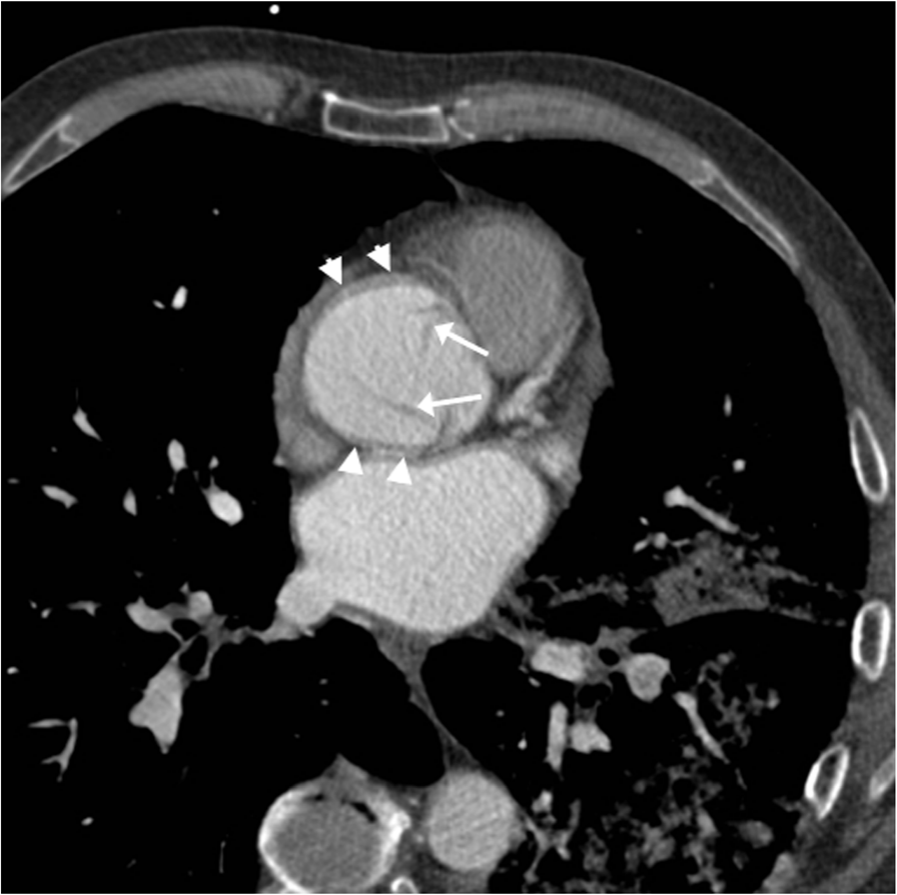
**Initial gated non-cardiac computed tomographic angiogram.** A gated non-cardiac axial contrast-enhanced computed tomographic scan of the sinuses of Valsalva is shown. Aortic motion artefact blurs the aortic wall anteriorly and posteriorly (arrowheads). This is the plane of aortic translocation during the cardiac cycle. The lines within the sinuses (arrows) were misinterpreted as aortic valve leaflets but lie above the cranial limit of normal leaflet tips.

**Figure 2:**
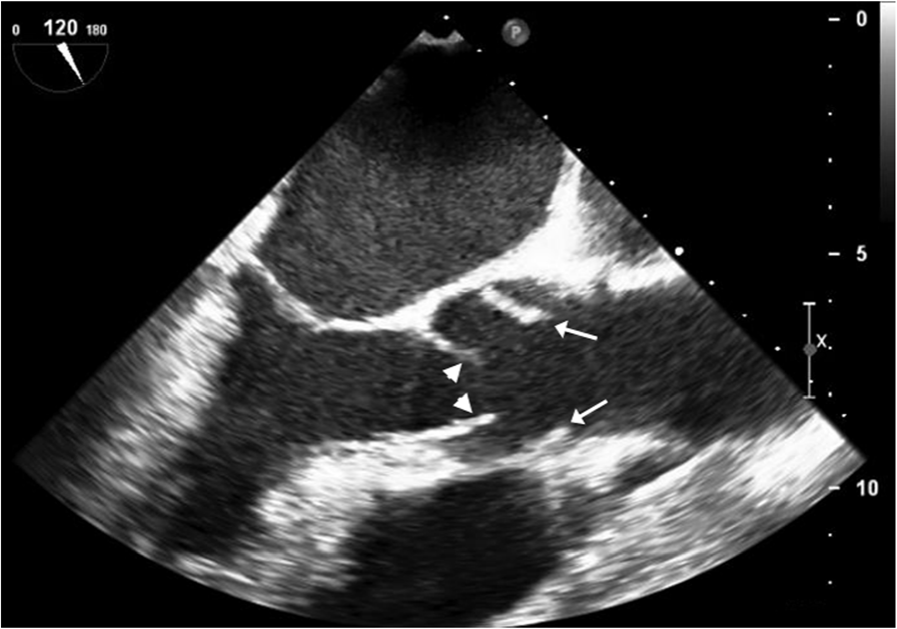
**Transesophageal echocardiogram.** Transesophageal echocardiographic scan of the aortic root showing the open leaflets of the aortic valve (arrowheads) and the dissection flap just above the valve and within the sinuses of Valsalva (arrows).

**Figure 3:**
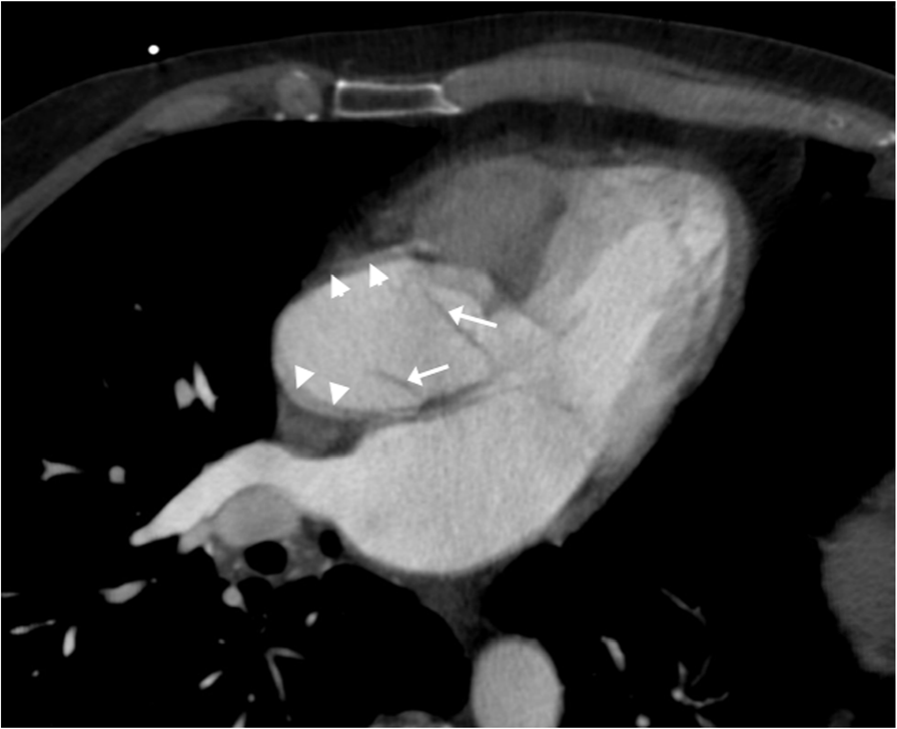
**Three-dimensional reformat of the original computed tomographic angiogram.** This three-dimensional reformat of the original contrast-enhanced computed tomographic scan shows the aortic root at an orientation similar to that of the transesophageal echocardiogram in Figure 2. Aortic motion artefact blurs the aortic wall (arrowheads). The lines above the aortic valve (arrows) cannot be explained by the valve leaflets and represent the dissection flap.

On the basis of the patient’s abrupt onset of symptoms, family history of aortic dissection and findings consistent with acute aortic insufficiency (large-volume pulse, early diastolic murmur and heart failure), an emergent contrast-enhanced CT scan was obtained (Figure 1). This scan showed a 5cm aortic root aneurysm but no aortic dissection. The patient was treated medically for acute coronary syndrome and heart failure, including dual anti-platelet therapy and anti-coagulation. He was subsequently transferred to a tertiary care hospital. Because of the high clinical suspicion of an acute aortic dissection based on his family history, sudden onset chest discomfort and findings of acute aortic insufficiency and heart failure [1], a transesophageal echocardiogram was taken, even in light of the negative CT scan (Figure 2). This test demonstrated an aortic dissection flap confined to the aortic root, superior to the aortic valve and associated with severe aortic insufficiency. The patient underwent successful repair of his Stanford type A aortic dissection with a 27mm mechanical valved conduit 21 hours after his initial presentation to the community hospital. The pathology report showed a structurally normal trileaflet native valve and cystic medial necrosis of the wall of the aortic root. Specialist cardiovascular radiologists who reviewed the initial CT scan *post hoc* reprocessed the raw data and correctly identified the dissection above the aortic valve (Figure 3). The dissection flap had initially been misinterpreted as a combination of normal aortic valve apparatus and motion artefact.

## Discussion

Early mortality in acute type A aortic dissection is high [2], and, although our patient did well, the initial false-negative CT scan delayed definitive treatment and exposed him to potentially harmful anti-coagulants. Some reports of CT scanning in acute dissection have quoted sensitivities of 100% [3,4], but false-negative scans are well-recognized [5,6]. True sensitivity is a little lower at 98% to 100% [7,8]. Conventional spiral CT angiography of the aortic root suffers from motion artefact, aortic translocation from cardiac motion, and pulsatility [9]. Subtle dissections limited to the aortic root and proximal aorta can be dismissed as artefacts. EKG-gated or triggered CT scans reduce motion artefacts and have been shown to improve diagnostic accuracy [10,11], but this imaging modality is not routinely used in all centers. Second imaging tests are frequently obtained in cases of suspected aortic dissection, with echocardiography, either transthoracic or transesophageal, being the most common second modalities used [1].

## Conclusion

Our present case is a reminder that the sensitivity of conventional CT angiography to rule out acute aortic syndromes is not 100% and supports the 2010 American College of Cardiology Foundation/American Heart Association Thoracic Aortic Disease Guidelines statement, which recommends that “if a high clinical suspicion exists for acute aortic dissection but initial aortic imaging is negative, a second imaging study should be obtained (Level of Evidence: C)” [5]. Cardiac gating of CT angiograms to exclude aortic dissection may improve diagnostic accuracy.

## Consent

Written informed consent was obtained from the patient for publication of this case report and any accompanying images. A copy of the written consent is available for review by the Editor-in-Chief of this journal.

## Abbreviations

CT: Computed tomographic; EKG: Electrocardiogram.

## Competing interests

No competing interests exist for any of the authors.

## Author’s contributions

KN and MSM wrote the case report and conducted the literature review. RC provided the images, including the three-dimensional reconstruction of the CT angiograms, and informed the interpretation of the images. All authors read and approved the final manuscript.
